# Recent Systemic Antifungal Exposure and Nonsusceptible *Candida* in Hospitalized Patients, South Africa, 2012–2017

**DOI:** 10.3201/eid3110.250359

**Published:** 2025-10

**Authors:** Charlotte Rabault, Liliwe Shuping, Ruth Mpembe, Vanessa Quan, Fanny Lanternier, Olivier Lortholary, Olivier Paccoud, Nelesh P. Govender, for GERMS-SA

**Affiliations:** National Institute for Communicable Diseases, Johannesburg, South Africa (C. Rabault, L. Shuping, R. Mpembe, V. Quan, N.P. Govender); Hôpital Universitaire Necker-Enfants malades Service des Maladies Infectieuses et Tropicales Adultes, Paris, France (C. Rabault, F. Lanternier, O. Lortholary, O. Paccoud); Institut Pasteur, Paris (F. Lanternier, O. Lortholary); Université Paris Cité, Paris (F. Lanternier, O. Lortholary ,O. Paccoud); University of the Witwatersrand Johannesburg Faculty of Health Sciences, Johannesburg (N.P. Govender); University of Cape Town Faculty of Health Sciences, Western Cape, South Africa (N.P. Govender); University of Exeter MRC Centre for Medical Mycology, Exeter, UK (N.P. Govender)

**Keywords:** Candida, antimicrobial resistance, fungi, antifungal drug resistance, antimicrobial stewardship, azoles, antifungal agents, fungal infections, South Africa

## Abstract

*Candida* bloodstream infections, and their increasing antifungal resistance, are a global concern. In this cross-sectional study, we analyzed 2,443 culture-confirmed candidemia cases reported in South Africa during 2012–2017 to assess the effect of previous antifungal exposure on nonsusceptible *Candida* infection. We classified cases by species resistance profile and patient’s antifungal use within 14 days before infection. We found that 48% of cases were caused by nonsusceptible species, and 20% of patients had prior antifungal exposure, mainly to fluconazole. In patients >90 days of age, prior antifungal use was significantly associated with nonsusceptible *Candida* bloodstream infection (adjusted OR 2.02, 95% CI 1.43–2.87; p<0.001), with species-specific effects. No such association was found in neonates and young infants, for whom hospital transmission appeared more influential. Our findings underscore the need for targeted antifungal stewardship and enhanced infection prevention to mitigate antifungal resistance in South Africa.

*Candida* bloodstream infections (BSIs) (i.e., candidemia) are among the most common invasive fungal infections globally, with an estimated 626,000 cases annually (*1*). Infections are associated with a substantial crude mortality rate of 35% (27%–60%) worldwide (*1*) and up to 44% in adults (*2*) and 38% in children (*3*) within South Africa. Since 2000, the epidemiology of *Candida* BSIs has shifted (*4*). *C. albicans*, which is largely fluconazole susceptible, has historically accounted for most infections, but it is now increasingly replaced by non-*albicans Candida* species (NAC), such as *Nakaseomyces glabratus* (formerly *C. glabrata*), that exhibit reduced susceptibility to >1 antifungal classes, either intrinsically or through acquisition of resistance mechanisms (*5*,*6*). 

South Africa is facing a large epidemic of BSI caused by antifungal-resistant (AFR) *Candida*. *C. parapsilosis* was documented as a leading cause of candidemia since 2009; more than two thirds of tested isolates exhibited resistance to azoles (*7*). *C. auris*, a multidrug-resistant species, has spread rapidly to become the second most common cause of candidemia since 2020 (*8*,*9*). The exact drivers of antifungal resistance in *Candida* BSIs remain inadequately understood. Prior exposure to antifungal agents has been described as a potent driver of NAC selection in settings with low resistance prevalence (*10*,*11*), in specific populations such as in intensive care units (ICU) (*12*), in hematologic and oncologic wards (*13*,*14*), or through ecologic studies (*15*). Such exposure has also been described as contributing to acquired resistance, mainly after prolonged therapy (*16*,*17*), but might not fully explain dissemination of those last strains (*18*,*19*), which has primarily been observed during outbreaks in local or regional healthcare settings (*20*–*22*). Most of those studies have focused on adult populations, with limited data from neonatal and pediatric populations, which account for most candidemia cases in South Africa (*8*). 

Treatment options are currently limited to 4 systemic antifungal classes: azoles, echinocandins, polyenes, and flucytosine (*23*). In low- and middle-income countries (LMIC), limited access to echinocandins, newer-generation azoles, and lipid amphotericin B formulations leads to a heavy reliance on fluconazole, despite the prevailing resistance patterns (*24*). Consequently, the effect of AFR is greater in those settings (*25*), leading to a higher risk for treatment failure (*26*) and use of toxic drugs such as conventional amphotericin B (*27*). Understanding factors leading to AFR is crucial to implement prevention or mitigation strategies (*28*). In this study, we aimed to determine whether recent exposure to systemic antifungal drugs was associated with the occurrence of nonsusceptible *Candida* BSI among hospitalized patients at sentinel sites in South Africa. 

## Materials and Methods

### Study Design and Surveillance Methods

In this cross-sectional study nested within the nationwide laboratory-based surveillance network in South Africa (GERMS-SA), we included all patients with culture-confirmed candidemia caused by any of the 6 most common *Candida* species (*C. albicans*, *N. glabratus*, *Pichia kudriavzevii* [formerly *C. krusei*], *C. auris*, *C. parapsilosis*, or *C. tropicalis*) identified in 31 hospital enhanced surveillance sites (ESS) in South Africa during January 1, 2012–December 31, 2017. GERMS-SA receives annual approvals from relevant university and provincial ethics committees in South Africa. We obtained additional ethics clearance from the Human Research Ethics Committee (Medical) of the University of Witwatersrand for this substudy (no. M240625).

The GERMS-SA surveillance methodology has been thoroughly described elsewhere (*7*,*8*). In brief, diagnostic laboratories at surveillance sites were requested to report all episodes of candidemia to the National Institute for Communicable Diseases (NICD; Johannesburg, South Africa). We defined candidemia as illness in a person from whom any *Candida* species was identified from a blood culture specimen. We defined an episode as a 30-day period starting from the date of the first positive *Candida* species culture. Isolation of a new and different *Candida* species within that 30-day period, or any subsequent positive blood cultures after the 30-day period, defined a recurrent episode and a new case. We considered isolation of multiple *Candida* species within the same blood culture set to be a mixed episode. Nurse surveillance officers at ESS prospectively collected additional clinical data using standardized case report forms. The number of ESS and coverage of surveillance expanded over the study period, from 9 ESS in 2 provinces in 2012–2013 to 16 ESS in 8 provinces in 2014–2015, and finally to 16 ESS as well as non-ESS from the public and private health sectors in 2016–2017. Case definitions and methodology remained consistent throughout the 3 study periods.

### Diagnostic Laboratory Practices

We confirmed species identification using the Vitek-2 system (bioMérieux, https://www.biomerieux.com) or matrix-assisted laser desorption/ionization time-of-flight mass spectrometry (Bruker Daltonics, https://www.bruker.com). We determined MICs of all tested agents except amphotericin B (fluconazole, voriconazole, itraconazole, posaconazole, micafungin, anidulafungin, caspofungin) by using commercial microbroth dilution panels containing Alamar blue (Thermo Fisher Scientific, https://www.thermofisher.com), read visually after 24 hours of incubation. We used *C. parapsilosis* ATCC 22019 and *P. kudriavzevii* ATCC 6258 as quality control isolates. We determined MICs of amphotericin B by Etest (bioMérieux) on RPMI-1640 plates containing 2% glucose (DMP, https://www.nhls.ac.za), as recommended by the manufacturer. For isolates with acquired resistance on microbroth dilution testing, we rechecked MICs by Etest. For species other than *C. auris*, we interpreted MICs using the available Clinical and Laboratory Standards Institute clinical breakpoints (*29*). For *C. auris*, we interpreted MICs using the US Centers for Disease Control and Prevention tentative clinical breakpoints (*30*).

### Definitions

We categorized episodes of candidemia into 2 groups on the basis of their identification and susceptibility profiles. We defined nonsusceptible *Candida* isolates as those exhibiting intrinsic nonsusceptibility (*C. auris*, *P. kudriavzevii*, and *N. glabratus*) or acquired resistance to >1 antifungal agent, classified as intermediate (I), susceptible-dose-dependent (SDD), or resistant (R). We chose that classification in the context of antifungal access in our setting, where azoles, particularly fluconazole, are the main first-line treatment for candidemia in the public sector. For mixed episodes, we considered only the most resistant isolate. We recorded prior systemic antifungal exposure to >1 antifungal agent (binary variable) within 14 days before the index blood culture collection date.

### Study Population and Participant Selection

We included only episodes with >1 antifungal agent viable isolate processed at the NICD reference laboratory for which we had species-level identification and antifungal susceptibility test results. We excluded recurrent episodes and patients missing data on the main exposure variable from the analysis.

### Data Management and Analysis

We used a probabilistic linking method (dtalink command on Stata [*31*]) for patient deduplication. We described patient characteristics using Fisher exact or χ^2^ tests for categorical variables and Student *t*-test or the Wilcoxon–Mann–Whitney test for continuous variables. To evaluate the effect of each confounder on the main association, we used a classical Mantel Haenszel method. We studied the association between prior antifungal exposure and nonsusceptible *Candida* BSI using a multivariable logistic regression adjusted for major confounders. Independence of individual participant outcomes could not be assumed because of observed and unobserved outbreaks leading to enhanced horizontal transmission within sites. Therefore, we integrated expected variation in candidemia risk factors and infection prevention and control measures at each ESS into the analysis and included a hospital site-level random effect regression analysis for multivariable analysis. To account for risk factors and antifungal prescription practices specific to neonates and young infants, as a prespecified effect modifier, we stratified our analysis by age group: neonates and young infants <90 days of age and patients >90 days of age. We excluded HIV status from the final models, despite its potential as a confounding factor, because the amount of missing data was substantial (>40%). We considered 2-sided p values of <0.05 significant. We performed all statistical analyses using Stata version 18 (StataCorp LLC, https://www.stata.com).

## Results

### Participants

After deduplication, we noted that 8,647 cases were detected during the 6-year surveillance period, of which 4,337 were nonrecurrent episodes and had both a confirmed identification for 1 of the 6 most prevalent *Candida* species and antifungal susceptibility test results. Of those, 2,443 patients were admitted to an ESS and had a completed case investigation form (Figure). We noted differences in age category, sex, year of diagnosis, healthcare sector, province of diagnosis, and *Candida* species between excluded and included cases (Appendix Table 1).

**Figure:**
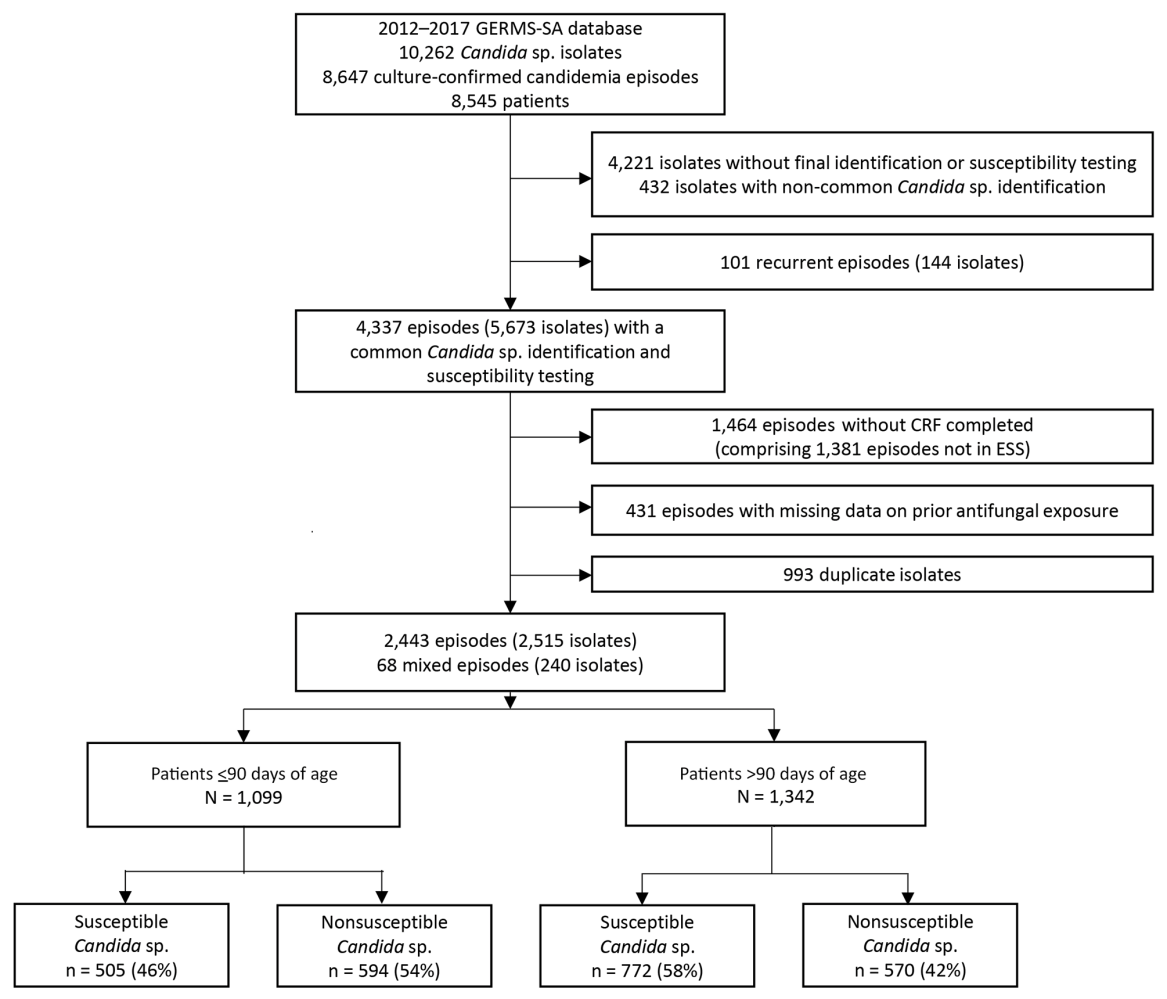
Flowchart of the selection process for 2,443 cases of candidemia from a 6-year surveillance period for secondary data analysis of recent systemic antifungal exposure and nonsusceptible *Candida* in hospitalized patients, South Africa, 2012–2017. Common *Candida* species were *C. albicans*, *C. parapsilosis*, *Nakaseomyces glabratus* (formerly *C. glabrata*), *C. auris*, *C. tropicalis*, and *Pichia kudriavzevii* (formerly *C. krusei*). CRF, case report form; ESS, enhanced surveillance site.

The population studied included 1,099 (45%) patients <90 days of age, comprising 831 neonates (<28 days) and 254 young infants (29–90 days of age), and 1,342 (55%) patients >90 days of age (Appendix Table 2). Overall, 94% (2,290/2,443) of patients were treated in a public-sector facility, and 71% (1,703/2,412) were admitted to an ICU. Most cases were recorded in Gauteng Province (1,397/2,443 cases [57%]) and during the 2016–2017 period (1,241/2,443 cases [51%]). Cases in neonates and young infants were reported more often than in patients >90 days of age during 2014–2015 (353/1,099 [32%] vs. 242/1,342 [18%]; p<0.001), in Gauteng Province (681/1,099 [62%] vs. 715/1,342 [53%]; p<0.001), and in the public sector (1,094/1,099 [99%] vs. 1,194/1,342 [89%]; p<0.001). Neonates and young infants were also more often hospitalized in an ICU (960/1,089 [88%] vs. 742/1,321 [56%]; p<0.001), had a longer median hospital stay before infection onset (median [IQR] 14 [9–22] vs. 11 [4–26] days; p<0.001), but less frequently had a central venous catheter in situ (551/1,078 [51%] vs. 742/1,321 [56%]; p = 0.001) or total parenteral nutrition administered (211/1,033 [20%] vs. 958/1,308 [24%]; p = 0.05).

### Prior Antifungal Exposure and Nonsusceptible *Candida* sp.

Overall, recent exposure to a systemic antifungal drug was recorded in 482/2,443 episodes (20%), in a larger proportion of neonates and young infants (272/1,099 [25%]) than older patients (210/1,342 [16%]; p<0.001). The most prescribed agent was fluconazole (340/482 [71%]), in similar proportions in the 2 age groups (196/272 [72%] in neonates and infants vs. 144/210 [69%] in older patients; p = 0.41), followed by amphotericin B deoxycholate (AmBD) (106/482 [22%]), in a higher proportion of neonates and young infants than patients >90 days of age (75/272 [28%] vs. 31/210 [15%]; p = 0.001). Echinocandins were prescribed for 48 patients, 5/272 (92%) for neonates and young infants versus 43/210 (20%) for patients >90 days of age (p<0.001). Exposure to multiple agents was recorded in 12% of patients (n = 60), mainly as a fluconazole and AmBD combination (Table 1; Appendix Table 3).

**Table 1:** Baseline characteristics by prior exposure to antifungal drugs in patients >90 days of age with culture-confirmed candidemia at sentinel hospitals, South Africa, 2012–2017*

Characteristic	No prior exposure, n = 1,132	Prior exposure, n = 210	All patients, N = 1,342	p value
Median age, y (IQR)	35 (8–55)	33 (3–54)	34 (8–55)	0.16†
Age group			1,342	
Older infants, 90 d–1 y	112 (10)	31 (15)	143	0.071
Children and adolescents, >1 y–17 y	198 (17)	40 (19)	238	
Adults, 18 y–64 y	663 (59)	105 (50)	768	
Elderly, >65 y	159 (14)	34 (16)	193	
Sex			1,341	
F	531 (47)	91 (43)	622	0.335
M	600 (53)	119 (57)	719	
Year of diagnosis			1,342	
2012–2013	288 (25)	63 (30)	351	<0.001
2014–2015	215 (19)	27 (13)	242	
2016	307 (27)	37 (18)	344	
2017	322 (28)	83 (40)	405	
Province§			1,342	
Gauteng	554 (49)	74 (35)	628	<0.001
Other	578 (51)	136 (65)	714	
Healthcare sector			1,342	
Private	99 (9)	49 (23)	148	<0.001
Public	1,033 (91)	161 (77)	1,194	
Prior hospitalization (<90 d)¶			1,328	
No	251 (22)	35 (17)	286	
Yes	869 (76)	173 (83)	1,042	
ICU admission			1,321	
No	511 (46)	68 (33)	579	<0.001
Yes	601 (54)	141 (67)	742	
Hospital stay before infection onset, d			1309	
Median (IQR)	10 (3–22)	23 (12–41)	11 (4–26)	<0.001†
<2	270 (25)	7 (3)	277	<0.001‡
3–7	191 (17)	23 (11)	214	
8–14	227 (21)	37 (18)	264	
15–21	129 (12)	31 (15)	160	
>22	285 (26)	109 (53)	394	
Systemic antimicrobial drug use			1,081	
No	342 (30)	31 (15)	373	<0.001
Yes	781 (70)	177 (85)	958	
Mechanical ventilation			1,316	
No	749 (68)	126 (61)	875	0.049
Yes	359 (32)	82 (39)	441	
CVC in situ			1,314	
No	505 (46)	51 (25)	556	<0.001
Yes	603 (54)	155 (75)	758	
TPN			1,308	
No	876 (79)	120 (59)	996	<0.001
Yes	230 (21)	82 (41)	312	
HIV status			771	
Seronegative	386 (59)	78 (68)	464	0.069
Seropositive	270 (41)	37 (32)	307	

*Values are no. (%) except as indicated. p value determined by Pearson χ^2^ test except as indicated. Numbers for each category indicate no. patients for whom that information was available. Prior antifungal exposure was <14 d before blood culture collection. CVC, central venous catheter; ICU, intensive care unit; TPN, total parenteral nutrition.  †p value determined by Wilcoxon rank-sum test. ‡p value determined by Fisher exact test. §Other province includes Limpopo, Mpumalanga, KwaZulu-Natal, Free State, North West, Northern Cape, Western Cape, Eastern Cape Province. ¶Prior hospital admission was recorded within the previous 90 d before current admission.

Of the 2,443 culture-confirmed candidemia cases, 1,165 were classified as nonsusceptible (48%), including 542 (47%) intrinsically nonsusceptible (*N. glabratus* 27%, *C. auris* 7%, *P. kudriavzevii* 13%) and 623 (53%) with acquired resistance (fluconazole-resistant *C. parapsilosis* 51%, fluconazole-resistant *C. albicans* 1%) (Appendix Table 6). Nonsusceptible *Candida* cases were found in a higher proportion of neonates and young infants than patients >90 days of age (594/1,099 [54%] vs. 772/1,342 [43%]; p<0.001).

### Effect of Prior Exposure to Antifungals on Nonsusceptible *Candida* BSI

The unadjusted odds ratio (OR) of nonsusceptible *Candida* candidemia among previously exposed neonates and young infants was 1.39 (95% CI 1.05–1.84) times higher than those nonexposed (Appendix Table 4). After adjusting for hospital site, age, sex, time period, province, type of delivery, and birthweight, OR decreased to 1.06 (95% CI 0.75–1.49) (Table 2). The final model revealed a strong cluster effect; differences between hospitals accounted for 14% of the variability in the occurrence of nonsusceptible *Candida* BSI (intracluster correlation coefficient = 0.14; p<0.001).

**Table 2:** Random-effect multivariable logistic regression analysis of the effect of prior exposure to antifungal drugs on nonsusceptible *Candida* bloodstream infections among patients with culture-confirmed candidemia, South Africa, 2012–2017*

Variable	<90 d of age†			>90 days of age‡	
	Summary aOR (95% CI) for nonsusceptible *Candida* spp.	Wald p value		Summary aOR (95% CI) for nonsusceptible *Candida* spp.	Wald p value
Prior antifungal exposure					
No	Referent			Referent	
Yes	1.06 (0.75–1.49)	0.73		2.02 (1.43–2.87)	<0.001
Age group					
Neonates, <28 d	Referent			NA	
Young infants, 29 d–90 d	0.79 (0.53–1.16)	0.23		NA	
Older infants, >90 d–1 y	NA			Referent	
Children–adolescents, >1 y–17 y	NA			0.95 (0.58–1.56)	0.84
Adults, 18 y–64 y	NA			1.92 (1.26–2.91)	0.002
Elderly, >65 y	NA-			2.84 (1.70–4.74)	<0.001
Sex					
F	Referent			Referent	
M	1.11 (0.84–1.47)	0.36		1.03 (0.81–1.32)	0.47
Year		0.02			
2012–2013	Referent			Referent	
2014–2015	1.97 (1.15–3.38)	0.01		1.06 (0.71–1.58)	0.77
2016	1.47 (0.92–2.32)	0.1		1.65 (1.18–2.32)	0.004
2017	1.82 (1.16–2.84)	0.009		1.49 (1.07–2.08)	0.02
Province					
Other	Referent			Referent	
Gauteng	2.93 (1.21–7.14)	0.01		1.64 (1.25–2.14)	<0.001
ICU admission					
No	NA			Referent	
Yes	NA			1.95 (1.51–2.54)	<0.001
Healthcare sector					
Private	NA			Referent	
Public	NA			0.36 (0.21–0.60)	<0.001
Hospital stay before infection onset, d					
<2	Referent			Referent	
3–7	1.47 (0.78–2.80)	0.24		0.71 (0.47–1.05)	0.09
8–14	1.86 (1.06–3.28)	0.03		0.77 (0.53–1.13)	0.19
15–21	2.41 (1.33–4.39)	0.004		0.79 (0.51–1.23)	0.29
>22	2.53 (1.38–4.64)	0.003		0.98 (0.69–1.40)	0.90
Type of delivery					
Cesarean section	Referent			NA	
Vaginal delivery	0.75 (0.56–1.00)	0.05		NA	
Birthweight		0.02			
NBW, >2,500 g	Referent			NA	
LBW, <2,500 g	1.32 (0.86–2.02)	0.21		NA	
VLBW, <1,500 g	1.49 (0.98–2.26)	0.06		NA	
ELBW, <1,000 g	1.82 (1.11–2.97)	0.018		NA	

*aOR, adjusted odds ratio; ELBW, extremely low birthweight; ICU, intensive care unit; LBW, low birthweight; NA, not applicable; NBW, normal birthweight; VLBW, very low birthweight. †23 clusters, mean observations per group = 42.3 (range 1–296); intracluster correlation coefficient = 0.14; p<0.001. ‡31 clusters, mean observations per group = 42.5 (range 1–166); intracluster correlation coefficient <0.0001; p = 0.5.

The unadjusted OR of nonsusceptible *Candida* BSI among previously exposed older patients was 2.23 (95% CI 1.66–3.01) times higher than those nonexposed (Appendix Table 5). After adjusting for hospital site, age, sex, time-period, province, ICU admission and healthcare sector, OR remained at 2.02 (95% CI 1.43–2.87) times higher in patients with a prior exposure compared with those without prior exposure (Table 2). We observed no meaningful cluster effect in the final model (intracluster correlation coefficient <1%; p = 0.5).

Among older patients, we observed an increased risk for nonsusceptible *Candida* BSI for those with prior exposure to azoles (unadjusted OR 1.89 [95% CI 1.35–2.64]) and echinocandins (unadjusted OR 6.75 [95% CI 3.10–14.69]) but not after prior exposure to AmBD (OR 1.87 [0.91–3.84]) (Appendix Table 8). Risks remained similar for each class after exclusion of cases with multiagent exposure (data not shown). We compared cases with and without prior exposure to azole; cases with recorded prior exposure to azole had a lower percentage of *C. albicans* (42/151 [28%]) than those without prior exposure (518/1,101 [47%]; p<0.001). We also noted an increase in *C. parapsilosis *in cases with prior azole exposure (54/151 [36%]) than cases without prior exposure (245/1,101 [22%]; p<0.001) (Table 3). We observed similar change in distribution after echinocandins exposure compared with no prior exposure for *C. albicans* (4/41 [10%] vs. 518/1,101 [47%]; p< 0.001) and *C. parapsilosis* (24/41 [59%] vs. 245/1,101 [22%]; p<0.001). The proportion of *C. auris* cases was statistically higher after azole prior exposure (16/151 [11%] vs. 44/1,101 [4%]; p = 0.002) than that of cases without prior exposure. Among *C. parapsilosis* cases, the proportion of fluconazole-nonsusceptible isolates significantly increased after exposure to echinocandins (22/24 [92%] vs. 121/245 [49%]; p<0.001), but not after azole exposure (32/54 [59%] vs. 121/245 [49%]; p = 0.19), compared with cases without exposure recorded.

**Table 3:** Comparaison of distribution of *Candida* isolates in patients >90 d of age (n = 1,301) with and without prior antifungal exposure, South Africa, 2012–2017*

Isolate	No prior exposure, n = 1,101	Prior exposure							
		To azoles, n = 151	p value		To echinocandins, n = 41	p value		To amphotericin B, n = 28	p value
*C. albicans*	518 (47)	42 (28)	<0.001		4 (10)	<0.001		6 (21)	ND
*Nakaseomyces glabratus*	202 (18)	20 (13)	0.09		1 (2)	ND/		1 (3)	ND
*C. auris*	44 (4)	16 (10)	<0.001		10 (24)	ND		9 (32)	ND
*C. parapsilosis*	245 (22)	54 (36)	<0.001		24 (59)	<0.001		8 (29)	ND
FLU-R	121 (11)	32 (21)	0.19		22 (54)	<0.001		2 (7)	ND
FLU-S	124 (11)	22 (15)	ND		2 (5)	ND		6 (21)	ND
*C. tropicalis*	57 (5)	8 (5)	0.97		2 (5)	ND		2 (7)	ND
*Pichia kudriavzevii*	34 (3)	11 (7)	0.005		0 (0)	ND		2 (7)	ND

*Values are no. (%) except as indicated. Cases with multiple exposures or mixed episodes are excluded. p values derived by Fisher exact test when validation conditions were met. FLU-R, fluconazole nonsusceptible; FLU-S, fluconazole susceptible; ND, not determined because of the lack of verification of the assumptions required for the statistical tests.

## Discussion

In a high AFR prevalence setting, we found that prior systemic antifungal exposure was independently associated with nonsusceptible *Candida* BSI in patients >90 days of age, but not among neonates and young infants (<90 days of age). Conversely, we observed a significant cluster effect exclusively among neonates and young infants, suggesting that the occurrence of nonsusceptible *Candida* BSIs was not independent in this population. Furthermore, in the older age group, the effect of prior antifungal exposure was drug- and species-specific; we observed a relatively higher proportion of *C. parapsilosis* and *C. auris* cases after azole and echinocandin preexposure, including a higher proportion of fluconazole-resistant *C. parapsilosis*, compared with cases without recorded prior antifungal exposure.

Previous studies showing an association between prior antifungal exposure and nonsusceptible *Candida* BSI in adults used heterogeneous definitions and often focused on specific antifungal–species combinations. A surveillance-based study in France that included 2,441 candidemia episodes showed that prior exposure to fluconazole within the previous 30 days was associated with increased odds (OR 2.17 [95% CI 1.51–3.13]) of candidemia caused by species with intrinsically reduced fluconazole susceptibility (mainly *N. glabratus* and *P. kudriavzevii*), whereas caspofungin preexposure was responsible for a relative increase in cases of *C. parapsilosis*, *N. glabratus,* and *P. kudriavzevii* compared with *C. albicans* (*11*). A study focusing on prior fluconazole exposure found an increased risk ratio of 4.47 (95% CI 2.12–9.43) of NAC BSI (*10*). Another study found that prior exposure to any type of antifungal agent was associated with an increased risk for fluconazole nonsusceptible *Candida* isolate (*32*). However, most of those studies included <400 episodes; few had >13% of *C. parapsilosis* compared with other species and almost no acquired resistance. Conversely, Shorr et al. (*33*) did not find an effect of prior exposure to fluconazole but used a broader definition of prior exposure (within the last 90 days) which might have diluted the effect. Blanchard et al. (*34*) found a significant positive association between prior exposure to echinocandins and reduced susceptibility to echinocandins in *Candida* spp. (OR 5.25; 95% CI 1.68–16.35). In this study, we used a broad definition of AFR taking into account both intrinsic and acquired resistance; acquired resistance increased recently, particularly in South Africa (*6*,*35*). Indeed, acquired resistance accounted for more than half of nonsusceptible episodes overall, largely because of fluconazole-resistant *C. parapsilosis* (95%). Although we also sought to assess echinocandin resistance, most of the nonsusceptibility patterns encountered in our study concerned azole resistance, and all echinocandin nonsusceptibility was detected among fluconazole–intrinsic resistance species. This is particularly worrisome when considering that fluconazole is one of the only accessible antifungal agents, along with amphotericin B deoxycholate, in the public sector, which covers ≈80% of the South Africa population. The low prevalence of echinocandin resistance observed can be linked to the relatively recent access to this antifungal class in the country; at the time of our study, it was available almost exclusively in the private sector. Similar to previous studies, we found a specific selection of *C. parapsilosis* after exposure to echinocandins, which can be explained by the intrinsically higher MICs of *C. parapsilosis* to echinocandins caused by a polymorphism in the *FKS* gene (*11*,*36*). The increased risk for *C. parapsilosis* after prior azole exposure was unexpected, particularly given the lack of observed differences between fluconazole-susceptible and -nonsusceptible isolates. Consistent with data from Vallabhaneni et al. (*18*), in which 59% of nonsusceptible *N. glabratus* cases had no known prior echinocandin exposure, the substantial proportion of nonsusceptible *Candida* BSI episodes without reported prior antifungal exposure suggests that additional factors may be contributing to antifungal resistance. 

We found no association between prior antifungal exposure and nonsusceptible *Candida* BSI among neonates and young infants. However, we observed a cluster effect that was not detected among patients >90 days of age. This finding suggests horizontal transmission during observed or unobserved outbreaks as the main mechanism of acquisition of nonsusceptible *Candida* strains in that population. Indeed, during the study period, the NICD investigated several large outbreaks of resistant *Candida* BSI in neonatal units (*37*). Studies focusing on the effect of antifungal prophylaxis on deaths in low-birthweight neonates did not identify an increased risk for nonsusceptible *Candida* strains (*38*).

Furthermore, we hypothesized that the differential impact of prior antifungal exposure could be attributed to a distinct baseline epidemiology with a higher proportion of *C. parapsilosis* and *P. kudriavzevii* in neonates and young infants. Therefore, the influence of prior exposure might be less pronounced compared with horizontal transmission. Most *P. kudriavzevii* isolates were linked to 2 outbreaks in a single neonatal unit in Gauteng Province during 2012–2015, in which overcrowding and suboptimal infection prevention and control practices were suggested as contributing factors despite the absence of a common environmental source (*8*). Since 2020, reports documenting the spread of *C. parapsilosis* clones in ICUs in the absence of prior antifungal exposure have increased (*20*); they were linked to invasive devices and found on healthcare workers’ hands (*39*). Furthermore, a specific clone of *C. parapsilosis* with acquired resistance to fluconazole, related to the *ERG11*p Y132F mutation, has been described globally (*21*,*40*) and in Gauteng Province (*41*); it is associated with persistent strains in the environment (*21*,*42*). Finally, the predominant use of polyenes as first-line treatment over azoles in the neonate population might also help explain the absence of observed resistance selection (*3*,*43*).

A limitation of this study is that it was not specifically designed to examine the association between prior antifungal exposure and nonsusceptible *Candida* BSI. We collected data on prior antifungal exposure using a binary question, completed by the type of antifungals but without details on indication (prophylaxis, preemptive, or empiric treatment), dosage, or length of exposure. Therefore, we cannot exclude the possibility that some of the recorded prior antifungal exposures were actually empiric or preemptive treatments, introducing a potential reverse causality bias. Our analysis was limited to antifungal exposure within the 14 days before the index culture, because of the structure of the case report forms. Further analyses exploring the effect of earlier exposures are warranted, particularly because a previous study (*44*) reported a higher risk for *N. glabratus* and *P. kudriavzevii* infections after >7 days of prior antifungal treatment compared with shorter treatment durations. Selection bias might be another limitation; we excluded 69% of cases diagnosed at sentinel hospitals because of missing data. Consequently, compared with the overall epidemiology of candidemia in the country, our sample included higher proportions of patients <18 years of age; cases caused by *C. albicans*, *C. tropicalis*, and *P. kudriavzevii*; cases from provinces other than Gauteng; and cases hospitalized in the public sector. The lack of a cluster effect observed in the older population may be attributed to the level (i.e., hospital facility) selected for the random effect; specifically, horizontal transmission appears to occur more frequently within wards than at the facility level. We excluded recurrent episodes from our analysis to avoid autocorrelation bias. However, we acknowledge that those episodes represent high-risk clinical scenarios for antifungal resistance. Dedicated analyses focusing on recurrent episodes are warranted and would contribute valuable insights into the role of prior antifungal exposure in the development of resistance. Finally, data regarding underlying conditions such as diabetes mellitus or immunocompromised status were poorly collected, resulting in residual confounding. We did not observe a change in point estimate for the main exposure effect in a smaller dataset comprising HIV status data (Appendix Table 9). Although those limitations may affect the generalizability of our findings, the large number of patients in our study provides important insights into potential mechanisms underlying AFR emergence and spread in South Africa.

Prior exposure to systemic antifungals appears to be a significant driver of nonsusceptible *Candida* BSI in patients >90 days of age. However, such exposure might not fully account for the epidemiologic patterns observed among neonates, where horizontal transmission may predominate. Neonates and young infants appear to be at particularly high risk of AFR in South Africa; specific studies should focus on that population. Finally, the implementation of antifungal stewardship programs must be accompanied by effective infection prevention and control policies to comprehensively address the large and complex issue of AFR in South Africa. 

Appendix Additional information about the association of recent systemic antifungal exposure and nonsusceptible *Candida* infections in hospitalized patients, South Africa, 2012–2017.
