# Supplementary material for: Recent Systemic Antifungal Exposure and Nonsusceptible Candida in Hospitalized Patients, South Africa, 2012–2017
Source: Emerg Infect Dis. 2025 Oct;31(10):1901–11. doi: 10.3201/eid3110.250359 (PMC12483017; doi:10.3201/eid3110.250359)
Supplement: Appendix — Additional information about the association of recent systemic antifungal exposure and nonsusceptible Candida infections in hospitalized patients, South Africa, 2012–2017. [file 25-0359-Techapp-s1.pdf]

Article DOI: https://doi.org/10.3201/eid3110.250359

EID cannot ensure accessibility for supplementary materials supplied by authors. Readers who have difficulty accessing supplementary content should contact the authors for assistance.

Recent Systemic Antifungal Exposure and Nonsusceptible *Candida* in Hospitalized Patients, South Africa, 2012–2017

Appendix

Supplementary Methods

Surveillance Methodology

At ESS, surveillance officers (SO, mainly trained nurses or pharmacists) prospectively collected additional clinical data for each case using standardised reports forms. This included information on antifungal use, HIV status, patient outcome and candidaemia risk factors, gathered through interviews with the case patient or next of kin, as well as hospital medical record review. Case patients were followed up only for the duration of their hospital admission. Additional case patients with laboratory-confirmed disease at NHLS laboratories, but not already reported to GERMS-SA, were audited to obtain basic demographic and laboratory data. In those cases, isolates were not available for identification confirmation. Data management was centralised at the NICD. Laboratory, clinical and demographic data from case patients were recorded on a Microsoft Access database. ESS were predominantly tertiary-level public healthcare facilities (except 3 private hospital sites in 2016–2017).

Study Population

We considered only the initial episode and excluded subsequent episodes to avoid autocorrelation. We also excluded patients with isolates that did not undergo susceptibility testing to avoid potential misclassification bias, as acquired resistance (only detected by susceptibility testing) constituted the primary resistance mechanism in our study population.

Variables, Confounding, and Interaction

The main explanatory variable was the patient's recent prior exposure to antifungals and which was recorded as a binary variable based on the response to the question: "Did the patients receive systemic antifungal medication in the 14 days before the date of positive culture was drawn? If yes, specify antifungals." Furthermore, when treatment variable was analyzed, all treatment started before 48hours before blood culture collection and stopped within the 14 days before blood culture collection was considered a recent prior exposure. Based on the relevant literature (*1*), we pre-selected key confounding factors from the variables recorded on the case report form. These selected risk factors for candidemia would likely prompt clinicians to prescribe antifungals and could also influence *Candida* colonization by facilitating the microorganism's proliferation. Therefore, for the neonates and young infant populations (≦90 days), the following variables were chosen: age group, sex, year of diagnosis, province, ICU admission, hospital stay before infection onset, type of delivery, CVC in situ, systemic antibiotic use, birthweight and type of feeding. Type of delivery is not traditionally considered a risk factor for candidemia or a justification for initiating antifungal treatment. However, contact with the birth canal during delivery has been shown to impact the microbiota of neonates (*2*) and facilitate the vertical transmission of multidrug-resistant bacteria, as previously reported (*3*,*4*). For the older population (>90 days), the following variable were chosen: age group, sex, year of diagnosis, healthcare sector of admission, ICU admission, hospital stay before infection onset, systemic antibiotic use, mechanical ventilation, CVC in situ, prior hospitalization (≦90 days), HIV status, total parenteral nutrition. The relationship between HIV status and the risk of candidemia caused by non-susceptible *Candida* has not yet been firmly established. However, given the high prevalence of this condition in South Africa (*5*), with a distinctive association with healthcare exposure (*6*) and antifungal prophylactic use (*7*,*8*), its impact was investigated through a subgroup analysis in the adult population. In both populations, even though sex is not a described confounding factor, it was kept in final models to consider unmeasured confounding. We classified potential confounding variables into clinically relevant categories. We accounted for epidemiologic temporal patterns by incorporating the 4-year period (2012–2013, 2014–2015; 2016; 2017), and for epidemiologic geographic factors and access to antifungal agents by adding the provinces as a binary variable (Gauteng vs Other, See Supplementary documents South Africa map) and the two healthcare sectors (public and private). Pre-term birth and birthweight

were categorized based on WHO definitions. None of the confounders were considered to be on the causal pathways for the main study question. We decided that the following potential confounding variables had collider relationship with the main exposure: ICU admission, CVC in situ and mechanical ventilation. ICU admission was used in our models. Similarly, pre-term birth and birthweight were considered to have a collider effect, and birthweight was used in our model.

Classical Analyses

We looked for evidence that the effect estimate for the association between prior exposition to antifungals and non-susceptible *Candida* BSI was confounded or modified by each explanatory variable. We compared the summary weighted odds ratio (OR) to the crude OR and looked for a ~10% change in main exposure effect estimate as evidence of confounding. We also visually compared the stratum-specific ORs across each level of the potential effect-modifying variable and performed a test of homogeneity of ORs.

Model Building

Independence of individual participant outcomes could not be assumed due to potent observed and unobserved outbreaks with enhanced horizontal transmission within one site. Therefore, variation in candidemia risk factors and infection prevention control measures at each sentinel site was integrated into the analysis using a center-level random effect regression analysis for multivariable analysis. We omitted potential confounders with small number of events collected. We forced all pre-specified confounder variables in the model. Reliability of estimates were checked using the quadchk command in Stata because the number of participants within many of the 31 clusters was large (>20).

Supplementary Results

Comparison of Prior Exposed and Non–Prior Exposed to Antifungal Patients

Aged $\leq$90 Days Group

Compared with 827 non exposed patient, a larger proportion of their exposed counterparts were older (median [IQR] age 22 days [15–31] versus 16 days [10–25]; $p < 0.001$), were hospitalized in Gauteng Province (130/272 [48%] versus 288/827 [34%]; $p < 0.001$) and in ICU ward (249/270 [92%] versus 711/819 [87%]; $p = 0.017$) and were more likely to present one

of the following risk factor of candidaemia: systemic antibiotic use (226/268 [84%] versus 565/819 [69%]; p < 0.001), mechanical ventilation (121/268 [45%] versus 247/813 [30%]; p < 0.001), CVC in situ (160/268 [60%] versus 419/391 [48%]; p = 0.001), a longer length of hospital stay before infection onset (median length of stay 19 days [14–29] versus 13 days [8–20]; p < 0.001) and a lower weight at birth (median [IQR] 1200 g [1000–1640] vs 1430 g [1090–2100]; p < 0.001). Exposed patients had been more delivered by caesarean compared to non-exposed patients (123/256 [48] vs 310/763 [41%], p < 0.001). No difference was observed regarding the sex, year of diagnosis and type of feeding (Appendix Table 3).

Aged >90 Days Group

Compared with 1332 non exposed patient, a larger proportion of their exposed counterparts were more hospitalized in the private sector (49/210 (23%) versus 99/1132 (9%); p < 0.001), in Gauteng province (136/210 (35%) versus 554/1132 (49%); p < 0.001) and in ICU ward (141/209 (67%) versus 601/1112 (54%); p < 0.001) and were more likely to present one of the following risk factor of candidaemia: systemic antibiotic use (177/208 (85%) versus 781/1123 (70%); p < 0.001), CVC in situ (155/206 (75%) versus 603/1108 (54%); p = 0.001), a longer length of hospital stay before infection onset (median length of stay 10 days [3–22] versus 23 days [12–41]; p < 0.001). No difference was observed regarding the age, sex, and HIV status (Table 1).

Unadjusted Risk Factors for Non-Susceptible *Candida* BSI

Aged ≤90 Days Group

The risk of non-susceptible *Candida* BSI followed a temporal and geographic repartition, with an increased risk from 2012 to 2017 (p = 0.04) and in Gauteng (OR 2.26 [1.76–2.89]; <0.001) compared to other provinces. This risk was also incrementally increased by the length of hospital stay before infection onset (p < 0.001). Admission in ICU ward increased the risk of 74% of non-susceptible *Candida* BSI (95%CI 1.20–2.53) compared with non-ICU wards. A vaginal delivery was protective against non-susceptible *Candida* BSI (OR 0.64 [0.5–0.83]; p < 0.001). The risk was incrementally decreased by the birthweight (p = 0.003). No other risk factors for candidaemia were associated with the risk of non-susceptible *Candida* BSI (Appendix Table 4).

Aged >90 Days Group

The risk of non-susceptible *Candida* BSI incrementally increased in each of the 3 older age groups compared with the baseline category (i.e., 90 days - 1-year-old) ($p < 0.001$). Similar to the other population, the risk followed a temporal and geographic repartition, with a higher risk in the more recent years (i.e., 2017; $p < 0.001$) and in Gauteng (OR 2.08 [1.66–2.59]; $p < 0.001$). Likewise, the risk was increased with the length of hospital stay before infection onset ($p < 0.001$) and admission in ICU ward (OR 2.60 [2.07–3.27]; $p < 0.001$). Being hospitalized in the public sector was associated with a 87% reduced risk, compared to the private sector (95%CI 0.08–0.20; $p < 0.001$). Common risk factors for candidaemia (i.e., systemic antibiotic use, mechanical ventilation, total parenteral nutrition and CVC in situ) were all associated with an increased risk of non-susceptible *Candida* BSI ($p < 0.05$). Among the 771 patients with available data, seropositivity to HIV was protective against non-susceptible *Candida* BSI (OR 0.61 [0.45–0.83]; p = 0,002). A prior hospitalization within the last 90 days was not associated with an increased risk of non-susceptible *Candida* BSI (Table 5).

Classical Stratified Analysis of the Relationship between Prior Antifungal Exposure and Non Susceptible Candida BSI

We confirmed our a-priori hypothesis regarding an interaction between prior antifungal exposure and age group on the overall population using a low- powered test for interaction (p = 0.02, <0.05) and visual inspection of the stratum-specific rate ratio (OR (95%CI) 1.37 [1.04–1.82] for neonates and young infants versus 2.28 [1,68–3.08] for older patients) (data not shown).

Aged ≦90 Days Group

Upon classical stratified analysis, the summary odds of non-susceptible Candida BSI remained positively associated (OR [95%CI] >1) among prior-exposed to antifungals patients compared to non-exposed patients, except for the type of delivery (OR (95%CI) 1.28 [0.95–1.70]), the length of hospital stay before infection onset (OR(95%CI) 1.24 [0.93–1.66]) and birthweight (OR(95%CI) 1.24 [0.93–1.66]). Province of admission was a negative confounder of the association between prior exposure and non-susceptible *Candida* BSI; the point estimate for the summary OR shifted by >10% from 1.39 [1.05–1.84] to 1.56 [1.17–2.08] after adjusting for this variable. Conversely, the summary OR point estimate shifted 10% lower to 1.25 [0.93–1.67]

after adjustment for birthweight and to 1.24 [0.93–1.66] after adjustment for length of hospital stay before infection onset. Using a low-powered test for interaction, we did not find evidence of an interaction between prior exposure to antifungals and the variables tested. Upon visual inspection, we identified the following as possible effect modifiers (different stratum-specific OR point estimates but overlapping 95%CI): Year of diagnosis, type of delivery and type of feeding. However, we did not analyze these as effect modifiers as we were already focusing on age group as an effect modifier because we had specified this a priori. The healthcare sector of admission was not included in bivariate and multivariable analysis for this population as only 4 patients were admitted in private sector facility (<10) (Appendix Table 6).

Aged >90 Days Group

Summary odds of non-susceptible Candida BSI remained ≈2-fold-higher among prior exposed to antifungals patients compared to non-exposed when adjusted for each potential confounder in turn. We found consistently strong evidence against the null hypothesis of an OR = 1 ($p < 0{,}001$ in all cases, except when adjusting for HIV status). Positive confounding on the main studied association with a shift >10% in the summary OR were observed for the following variables: age group, healthcare sector of admission, length of hospital stay before infection onset and HIV status. For the latter, a large amount of missing data was noted, thus the variable was not included in the main analysis but a reduced model was explored. Using a low-powered test for interaction, we found evidence of an interaction between prior exposure to antifungals and ICU admission ($p = 0.003$). Upon visual inspection, we identified the following as possible effect modifiers: age group, sex, healthcare sector of admission, year of diagnosis, province, length of hospital stay before infection onset, and prior antibiotics. Similar to the aged ≦90 days group, we did not analyze these as effect modifiers (Appendix Table 7).

Effect of prior exposure to antifungals on the proportion of non-susceptible strain candidaemia in older patients (>90 days) when including the HIV variable

When this variable was retained in a model with fewer observations (n = 762), the point estimate for the effect of prior antifungal exposure on non-susceptible *Candida* BSI was very similar to that described in the final model, but with a broader confident interval (Appendix Table 9).

References

1. Lass-Flörl C, Kanj SS, Govender NP, Thompson GR III, Ostrosky-Zeichner L, Govrins MA. Invasive candidiasis. Nat Rev Dis Primers. 2024;10:20. PubMed https://doi.org/10.1038/s41572-024-00503-3

2. Lai C, Huang L, Wang Y, Huang C, Luo Y, Qin X, et al. Effect of different delivery modes on intestinal microbiota and immune function of neonates. Sci Rep. 2024;14:17452. PubMed https://doi.org/10.1038/s41598-024-68599-x

3. Herindrainy P, Rabenandrasana MAN, Andrianirina ZZ, Rakotoarimanana FMJ, Padget M, de Lauzanne A, et al.; BIRDY study group. Acquisition of extended spectrum beta-lactamase-producing enterobacteriaceae in neonates: a community based cohort in Madagascar. PLoS One. 2018;13:e0193325. PubMed https://doi.org/10.1371/journal.pone.0193325

4. Chereau F, Herindrainy P, Garin B, Huynh BT, Randrianirina F, Padget M, et al. Colonization of extended-spectrum-β-lactamase- and NDM-1-producing Enterobacteriaceae among pregnant women in the community in a low-income country: a potential reservoir for transmission of multiresistant Enterobacteriaceae to neonates. Antimicrob Agents Chemother. 2015;59:3652–5. PubMed https://doi.org/10.1128/AAC.00029-15

5. Satoh S, Boyer E. HIV in South Africa. Lancet. 2019;394:467. PubMed https://doi.org/10.1016/S0140-6736(19)31634-4

6. Govender NP, Todd J, Nel J, Mer M, Karstaedt A, Cohen C, et al. HIV infection as risk factor for death among hospitalized persons with candidemia, South Africa, 2012–2017. Emerg Infect Dis. 2021;27:1607–15. https://wwwnc.cdc.gov/eid/article/27/6/21-0128_article.htm

7. Lortholary O, Petrikkos G, Akova M, Arendrup MC, Arikan-Akdagli S, Bassetti M, et al.; ESCMID Fungal Infection Study Group. ESCMID* guideline for the diagnosis and management of *Candida* diseases 2012: patients with HIV infection or AIDS. Clin Microbiol Infect. 2012;18(Suppl 7):68–77. PubMed https://doi.org/10.1111/1469-0691.12042

8. Chang CC, Harrison TS, Bicanic TA, Chayakulkeeree M, Sorrell TC, Warris A, et al. Global guideline for the diagnosis and management of cryptococcosis: an initiative of the ECMM and ISHAM in cooperation with the ASM. Lancet Infect Dis. 2024;24:e495–512. PubMed https://doi.org/10.1016/S1473-3099(23)00731-4

Appendix Table 1. Characteristics of 4,337 patients with candidaemia due to common *Candida* sp. at sentinel site who were included or excluded from analysis of prior exposure to antifungal as risk factor of non-susceptible *Candida* sp. bloodstream infection, South Africa, 2012–2017*

Characteristic	Total	No CRF completed/no ESS	Incomplete CRF on prior atf variable	Included
Total	4,337	1,464 (34)	`	2,442 (56)
Age group				
Neonates (<28 d)	1,130	141 (12)	159 (14)	831 (73)
Young infants (>28 d–90 d)	351	60 (17)	37 (11)	254 (68)
Older infants (>90 d–1 y)	230	54 (23)	33 (14)	143 (62)
Children – adolescents (>1 y–17 y)	352	78 (22)	36 (10)	238 (68)
Adults (18 y–64 y)	1,574	696 (44)	110 (7)	768 (49)
Elderly (>65 y)	621	398 (27)	30 (5)	193 (31)
missing	79	37 (47)	26 (33)	16 (20)
Sex				
F	1,960	788 (40)	204 (10)	1,111 (57)
M	2,325	645 (28)	214 (9)	1,324 (57)
missing	52	31 (60)	13 (25)	8 (15)
Healthcare sector of admission				
Public	3,122	408 (13)	425 (14)	2290 (73)
Private	1,215	1,056 (87)	6 (0)	153 (13)
Year of diagnosis				
2012–2013	826	0 (0)	219 (29)	607 (71)
2014–2015	645	14 (2.2)	36 (9)	595 (90)
2016	1,325	661 (50)	86 (6)	578 (44)
2017	1,541	789 (51)	90 (6)	663 (43)
Province				
Eastern Cape	115	18 (16)	6 (5)	91 (79)
Free State	316	28 (9)	13 (4)	275 (87)
Gauteng	2,733	967 (35)	370 (14)	1,397 (51)
KwaZulu-Natal	437	87 (20)	16 (4)	334 (76)
Limpopo	56	30 (54)	3 (5)	23 (41)
Mpumalanga	87	48 (55)	2 (2)	37 (43)
Northern Cape	37	7 (19)	1 (3)	29 (78)
North West	70	25 (36)	4 (6)	41 (59)
Western Cape	486	254 (52)	16 (3)	216 (44)
Candida species				
Candida albicans	1,507	355 (24)	164 (11)	989 (66)
Candida glabrata	552	179 (32)	55 (10)	318 (58)
Candida auris	334	235 (70)	13 (4)	86 (26)
Candida parapsilosis	1,714	669 (39)	167 (10)	878 (51)
Candida tropicalis	145	29 (20)	26 (20)	90 (62)
Pichia kudriavzevii	199	35 (18)	13 (7)	151 (76)

*Values are no. (%).

Appendix Table 2. Comparison of baseline characteristics by age group among 2,443 patients with culture-confirmed candidemia at enhanced surveillance sites, South Africa, 2012–2017*

Characteristic	No. patients† N = 2,443	Aged >90 d, n = 1,342	Aged ≤90 d, N = 1,099	p value‡
Median age, y (IQR)	1 (0–38)	34 (8–55)	(d) 18 (11–27)	
Age group	2,427			
Neonates (≤28 d)		NA	831 (34)	
Young infants (>28 d–90 d)		NA	254 (10)	
Older infants (>90 d–1 y)		143 (6)	NA	
Children – adolescents (>1 y–17 y)		238 (10)	NA	
Adults (18 y–64 y)		768 (32)	NA	
Elderly (≥65 y)		193 (8)	NA	
Sex	2,435			
F	1,111	622 (46)	489 (45)	0.430
M	1,324	719 (54)	603 (55)	
Year of diagnosis	2,443			
2012–2013	607	351 (26)	255 (23)	<0.001
2014–2015	595	242 (18)	353 (32)	
2016	578	344 (26)	234 (21)	
2017	663	405 (30)	257 (23)	
Province	2,443			
Other¶	1,046	628 (47)	418 (38)	<0.001
Gauteng	1,397	714 (53)	681 (62)	
Healthcare sector of admission	2,443			
Private	153	148 (11)	4 (0.3)	<0.001
Public	2,290	1,194 (89)	1,095 (99)	
ICU admission during hospital stay	2,412			
No	709	579 (44)	129 (12)	<0.001
Yes	1,703	742 (56)	960 (88)	
Median hospital stay before infection onset, d (IQR)	13 (6–24)	11 (–26) 4	14 (9–22)	<0.001§
Hospital stay before infection onset, d				
≤2	369	277 (21)	92 (8)	<0.001
3–7	340	214 (16)	126 (12)	
9–14	595	264 (20)	330 (30)	
15–21	404	160 (12)	244 (22)	
≥22	693	394 (30)	298 (27)	
Systemic antibiotic use	2,420			
No	669	373 (28)	296 (27)	0.665
Yes	1,751	958 (72)	791 (73)	
Mechanical ventilation	2,399			
No	1,590	875 (66)	713 (66)	0.784
Yes	809	441 (34)	368 (34)	
CVC in situ	2,394			
No	1,084	556 (42)	527 (49)	0.001
Yes	1,310	758 (58)	551 (51)	
Total parenteral nutrition	2,341			
No	1,818	996 (76)	822 (81)	0,05
Yes	523	312 (24)	211 (19)	
Prior hospitalization (≦90 d)		1,328	NA	
No		286 (22)	NA	
Yes		1,042 (78)	NA	
HIV status		771	NA	
Positive		464 (60)	NA	
Negative		307 (40)	NA	
Type of delivery		NA	1,019	
Caesarean section		NA	433 (42)	
Vaginal delivery		NA	586 (58)	
Median pre-term birth, w (IQR)			31 (28–34)	
Pre-term birth, weeks		NA	1,031	
≤28		NA	303 (29)	
29–32		NA	338 (33)	
33–37		NA	251 (24)	
≥38		NA	139 (13)	
Median birthweight, g (IQR)		NA	1,350 (1,070–2,030)	
Birthweight		NA	1,049	
NBW (≧2,500 g)		NA	178 (13)	
LBW (<2,500 g)		NA	267 (20)	
VLBW (<1,500 g)		NA	418 (31)	
ELBW (<1,000 g)		NA	186 (14)	

Characteristic	No. patients† N = 2,443	Aged >90 d, n = 1,342	Aged <90 d, N = 1,099	p value‡
Exposure to prior antifungals (≦14 d)	2,443			
No	1,961	1,132 (84)	827 (75)	<0.001
Yes	482	210 (16)	272 (25)	
Prior azole	365	158 (75)#	207 (76)#	0.83**
Prior echinocandins	48	43 (20)#	5 (2)#	<0.001**
Prior amphotericin B deoxycholate	106	31 (15)#	75 (28)#	<0.001**
Candida species	2,443			
C. albicans	954	570 (42)	384 (35)	<0.001
N. glabratus	311	240 (18)	71 (6)	
C. auris	82	80 (6)	2 (0.1)	
C. parapsilosis	862	331 (25)	531 (48)	
C. tropicalis	83	72 (5)	11 (1)	
P. kudriavzevii	149	49 (4)	100 (9)	

*Values are no. (%). Prior antifungal exposure was recorded within the previous 14 d before blood culture collection. Prior hospital admission was recorded within the previous 90 d before current admission. CVC, central venous catheter; ICU, intensive care unit; NA, not applicable.
†Total numbers for each category indicate number of patients for whom that information was available. For 2 patients, age group value is missing.
‡p values by Pearson's χ2 test unless otherwise indicated.
§By Wilcoxon rank-sum test.
¶Other Province: Limpopo, Mpumalanga, KwaZulu-Natal, Free State, North West, Northern Cape, Western Cape, Eastern Cape Province
#Percentages calculated from the total number of exposed patients may not add up to 100% due to multiple exposures.
**vs. exposure to other classes of antifungals.

Appendix Table 3. Comparison of baseline characteristics between exposed and non-exposed to prior antifungals among 1,099 neonates and young infants (<90 d) with culture-confirmed candidemia at sentinel hospitals, South Africa, 2012–2017*

Characteristic	No prior exposure, n (%), n = 827	Prior exposure, n (%), n = 272	No. patients, N = 1,099	p value†
Median age, d(IQR)	16 (10–25)	22 (15–32)	18 (11–27)	<0.001‡
Age group			1,085	
Neonates (≦28 d)	648 (79)	183 (68)	831	<0.001
Young infants (28d-90d)	169 (21)	85 (32)	254	
Sex			1,092	
F	372 (45)	117 (43)	489	0.625
M	451 (55)	152 (57)	603	
Year of diagnosis			1,099	
2012–2013	201 (24)	54 (20)	255	0.237
2014–2015	264 (32)	89 (33)	353	
2016	179 (22)	55 (20)	234	
2017	183 (22)	74 (27)	257	
Province			1,099	
Gauteng	288 (35)	130 (48)	418	<0.001
Other¶	539 (65)	142 (52)	681	
Health-care sector of admission			1,099	
Private	1 (0.1)	3 (0.1)	4	
Public	826 (99)	269 (99)	1,095	
Type of delivery			1,019	
Cesarean section	310 (41)	123 (48)	433	0.038
Vaginal delivery	453 (59)	133 (52)	586	
ICU admission			1,089	
No	108 (13)	21 (8)	129	0.017§
Yes	711 (87)	249 (92)	960	
Hospital stay before infection onset, d			1,090	
Median (IQR)	13 (8–20)	19 (14–29)	14 (9–22)	<0.001‡ †
<2	88 (11)	4 (1)	92	<0.001§
3–7	107 (13)	19 (7)	126	
8–14	273 (33)	57 (21)	330	
15–21	167 (20)	77 (28)	244	
>22	184 (22)	114 (42)	298	
Systemic antibiotic use			1,087	
No	254 (31)	42 (16)	296	<0.001
Yes	565 (69)	226 (84)	791	
Mechanical ventilation			1,081	
No	566 (70)	147 (55)	713	<0.001
Yes	247 (30)	121 (45)	368	
CVC in situ			1,078	

Characteristic	No prior exposure, n (%), n = 827	Prior exposure, n (%), n = 272	No. patients, N = 1,099	p value†
No	419 (52)	108 (40)	527	0.001
Yes	391 (48)	160 (60)	551	
Type of feeding			1,033	
Breast feeding	448 (58)	147 (57)	595	0.476
Formula/mixed	176 (23)	51 (20)	227	
Total parenteral nutrition only	153 (20)	58 (23)	211	
Pre-term birth, w			1,031	
Median (IQR)	31 (28–35)	30 (28–33)	31 (28–34)	<0.001‡
≤28	198 (26)	105 (41)	303	<0.001§
29–32	250 (32)	88 (34)	338	
33–37	205 (27)	46 (18)	251	
		))		
>38	120 (16)	19 (7)	139	
Birthweight, g			1,049	
Median (IQR)	1,430 (1,090–2,100)	1,200 (1,000–1,640)		<0.001‡
NBW (≥2,500 g)	150 (19)	28 (11)	178	<0.001§
LBW (<2,500 g)	218 (28)	49 (19)	267	
VLBW (<1,500 g)	298 (38)	120 (46)	418	
ELBW (<1,000 g)	122 (15)	64 (25)	186	

*Total numbers for each category indicate number of patients for whom that information was available. Prior antifungal exposure was recorded within the previous 14 d before blood culture collection. CVC, central venous catheter; ICU, intensive care unit.
†p values by Pearson's χ^2 test unless otherwise indicated.
‡ By Wilcoxon rank-sum test.
§By Fisher exact test.
¶Other province: Limpopo, Mpumalanga, KwaZulu-Natal, Free State, North West, Northern Cape, Western Cape, Eastern Cape Province.

Appendix Table 4. Risk factors for non-susceptible *Candida* sp. bloodstream infection among 1,099 neonates and young infants (≤90 d) patients with culture-confirmed candidemia at sentinel hospitals, South Africa, 2012–2017

Risk factor	Susceptible *Candida* sp., n (%) 505 (46%)	Nonsusceptible *Candida* sp., n (%) 594 (54%)	No. patients, N = 1,099	Crude OR (95% CI)	LRT p value
Prior exposure to antifungals			1,099		
No	396 (78)	431 (73)	827	Referent	0.02
Yes	109 (22)	163 (27)	272	1.39 (1.05–1.84)	
Age group			1,085		
Neonates (≤28 d)	373 (75)	458 (78)	831	Referent	0.19
Young infants (>28 d–90 d)	126 (25)	128 (22)	254	0.83 (0.62–1.10)	
Sex			1,092		
F	231 (46)	258 (44)	489	Referent	0.48
M	272 (54)	331 (56)	603	1,08 (0,86–1,38)	
Year of diagnosis			1,093		
2012–2013	130 (26)	125 (21)	255	Referent	0.04
2014–2015	161 (32)	192 (32)	353	1.24 (0.90–1.71)	
2016	114 (23)	120 (20)	234	1.09 (0.77–1.56)	
2017	100 (20)	157 (26)	257	1.63 (1.15–2.32)	
Province of hospitalization			1,099		
Gauteng	261 (52)	420 (71)	681	2.26 (1.76–2.89)	<0.001
Other Province	244 (48)	174 (29)	418	Referent	
Health-care sector of admission			1,099		
Private	0 (0)	4 (0.1)	4	ND	
Public	505 (100)	590 (99)	1,095	ND	
Type of delivery			1,019		
Vaginal	299 (63)	287 (53)	586	0.64 (0.50–0.83)	<0.001
Cesarean	174 (37)	259 (47)	433	Referent	
ICU admission			1,089		
No	75 (15)	54 (9)	129	Referent	0.03
Yes	426 (85)	534 (91)	960	1.74 (1.20–2.53)	
Hospital stay before infection onset, d			1,090		
≤2	59 (12)	33 (6)	92	Referent	0.002
3–7	62 (12)	64 (11)	126	1.85 (1.06–3.20)	
8–14	155 (31)	175 (30)	330	2.02 (1.25–3.25)	
15–21	103 (21)	141 (24)	244	2.45 (1.50–4.02)	

Risk factor	Susceptible *Candida* sp., n (%) 505 (46%)	Nonsusceptible *Candida* sp., n (%) 594 (54%)	No. patients, N = 1,099	Crude OR (95% CI)	LRT p value
≥22	123 (25)	175 (30)	298	2.54 (1.57–4.13)	
Systemic antibiotic use			1,087		
No	130 (26)	166 (28)	296	Referent	0.42
Yes	369 (74)	422 (72)	791	0.90 (0.68–1.17)	
Mechanical ventilation			1,081		
No	329 (66)	384 (66)	713	Referent	0.75
Yes	166 (34)	202 (34)	368	1.04 (0.81–1.34)	
CVC in situ			1,078		
No	244 (49)	283 (49)	527	Referent	0.86
Yes	252 (51)	299 (51)	551	1.02 (0.81–1.30)	
Type of feeding			1,033		
Breast feeding	270 (56)	325 (59)	595	Referent	0.51
Formula/mixed	113 (24)	114 (21)	227	0.84 (0.62–1.14)	
Total parenteral nutrition only	96 (20)	115 (21)	211	1.00 (0.73–1.36)	
Birthweight			1,049		
NBW (≥2,500 g)	97 (20)	81 (14)	178	Referent	0.03
LBW (<2,500 g)	123 (26)	144 (25)	267	1.40 (0.96–2.05)	
VLBW (<1,500 g)	180 (38)	238 (41)	418	1.58 (1.11–2.25)	
ELBW (<1,000 g)	74 (16)	112 (19)	186	1.81 (1.20–2.75)	

* ELBW, extremely low birthweight; LBW, low birthweight; NBW, normal birthweight; ND, not determined; VLBW, very low birthweight.

Appendix Table 5. Risk factors for non-susceptible *Candida* sp. bloodstream infection among 1332 old infants and older (>90 d) patients with culture-confirmed candidemia at sentinel hospitals, South Africa, 2012–2017

Risk factor	Susceptible *Candida* sp., n (%) N = 772 (58%)	Non susceptible *Candida* sp., n (%) N = 570 (42%)	No. patient N = 1,342	Crude OR (95% CI)	LRT p value
Prior exposure to antifungals			1,342		
No	687 (89)	445 (78)	1,132	Referent	<0.001
Yes	85 (11)	125 (22)	210	**2.23 (1.66–3.01)**	
Age group			1,342		
Older infants (>90d–1y)	102 (13)	41 (7)	143	Referent	<0.001
Children – adolescents (>1y–17y)	176 (23)	62 (11)	238	0.88 (0.55–1.39)	
Adults (18y–64y)	418 (54)	350 (61)	768	2.08 (1.41–3.07)	
Elderly (≧65y)	76 (10)	117 (21)	193	3.83 (2.41–6.09)	
Sex			1,341		
HM	200 (35)	319 (56)	719	1.18 (0.95–1.47)	0.14
F	371 (65)	251 (44)	622	Referent	
Year of diagnosis			1,342		
2012–2013	225 (29)	126 (22)	351	Referent	<0.001
2014–2015	173 (22)	69 (12)	242	0.71 (0.50–1.01)	
2016	180 (23)	164 (29)	344	1.63 (1.20–2.20)	
2017	194 (25)	211 (37)	405	1.94 (1.45–2.60)	
Province of hospitalization			1,342		
Gauteng	352 (46)	362 (64)	714	2.08 (1.66–2.59)	<0.001
Other province*	420 (54)	208 (36)	628	Referent	
Health-care sector			1,342		
Private	26 (3)	122 (21)	148	Referent	
Public	746 (97)	448 (79)	1,194	0.13 (0.08–0.20)	<0,001
Prior hospitalization			1,328		
No	178 (23)	108 (19)	286	Referent	0,07
Yes	587 (77)	455 (81)	1,042	1.28 (0.98–1.67)	
ICU admission			1,321		
No	408 (53)	171 (31)	579	Referent	<0,001
Yes	355 (47)	387 (69)	957	2.60 (2.07–3.27)	
Hospital stay before infection onset, d			1,309		
≤ 2	172 (23)	105 (19)	277	Referent	<0.001
3–7	137 (18)	77 (14)	214	0.92 (0.64–2.33)	
8–14	155 (21)	109 (19)	264	1.15 (0.82–1.63)	
15–21	95 (13)	65 (12)	160	1.12 (0.75–1.67)	
≥22	191 (25)	203 (36)	394	1.74 (1.27–2.38)	

Risk factor	Susceptible *Candida* sp., n (%) N = 772 (58%)	Non susceptible *Candida* sp., n (%) N = 570 (42%)	No. patient N = 1,342	Crude OR (95% CI)	LRT p value
Systemic antibiotic use			1,081		0.04
No	232 (30)	141 (25)	373	Referent	
Yes	536 (70)	422 (75)	958	1.30 (1.01–1.66)	
Mechanical ventilation			1,316		
No	545 (71)	330 (60)	875	Referent	<0,001
Yes	220 (29)	221 (40)	441	1.66 (1.32–2.09)	
CVC in situ			1,314		
No	360 (47)	196 (35)	556	Referent	<0,001
Yes	398 (53)	360 (65)	758	1.66 (1.33–2.08)	
TPN			1,308		
No	615 (81)	381 (70)	996	Referent	<0,001
Yes	146 (19)	166 (30)	312	1.83 (1.42–2.37)	
HIV status			771		
Seronegative	270 (56)	194 (67)	464	Referent	0,002
Seropositive	213 (44)	94 (33)	307	0.61 (0.45–0.83)	

*Other province: Limpopo, Mpumalanga, KwaZulu-Natal, Free State, North West, Northern Cape, Western Cape, Eastern Cape. Province.

Appendix Table 6. Effect of 14 d prior exposure to antifungals on non-susceptible *Candida* sp. bloodstream infection among 1,099 neonates and young infants (≦90 d) patients, adjusted in turn for each potential confounder, South Africa, 2012–2017*

Variable	No. patients	Stratum-specific OR (95% CI)	Mantel-Haenszel summary OR (95% CI)	Score test p value	p value for test of interaction
Age group *(m = 14)*			1.40 (1.05–1.85)	0.02	0.26
≤28 days	831	1.54 (1.10–2.18)			
>28 d-90	254	1.09 (0.64–1.83)			
Sex *(m = 7)*			1.35 (1.02–1.78)	<0.04	0.34
F	489	1.16 (0.76–1.76)			
M	603	1.52 (1.04–2.22)			
Year *(m = 0)*			1.34 (1.02–1.78)	0.04	0.20
2012–2013		0.96 (0.52–1.75)			
2014–2015		2.07 (1.24–3.45)			
2016		1.31 (0.71–2.40)			
2017		1.07 (0.61–1.86)			
Province *(m = 0)*			1.56 (1.17–2.08)	0.002	0.37
Gauteng	681	1.38 (0.94–2.05)			
Other†	418	1.80 (1.18–2.75)			
ICU admission *(m = 10)*			1.35 (1.02–1.79)	<0.04	0.97
No	129	1.32 (0.51–3.40)			
Yes	960	1.35 (1.00–1.81)			
Length of hospital stay before infection onset *(m = 9)**					
≤2d	92	/	1.24 (0.93–1.66)	0.13	0.11
3–7d	126	1.81 (0.66–5.01)			
8–14d	330	1.38 (0.77–2.48)			
15–24d	244	0.96 (0.56–1.66)			
>24d	298	1.13 (0.70–1.82)			
Type of delivery *(m = 80)*			1.28 (0.95–1.70)	0.10	0.11
Caesarean section	433	1.66 (1.07–2.59)			
Vaginal delivery	586	1.03 (0.70–1.52)			
Prior antibiotics *(m = 12)*					
No	296	1.68 (0.84–3.36)	1.42 (1.07–1.88)	0.02	0.59
Yes	791	1.37 (1.00–1.87)			
Birthweight *(m = 58)**			1.23 (0.93–1.66)	0.15	0.48
NBW (≥2,500 g)	178	0.74 (0.32–1.69)			
LBW (<2,500 g)	267	1.17 (0.62–2.19)			
VLBW (<1,500 g)	418	1.52 (0.98–2.36)			
ELBW (<1,000 g)	186	1.16 (0.62–2.16)			
Type of feeding *(m = 66)*			1.39 (1.04–1.85)	0.03	0.05
Breastfeeding	595	1.43 (0.98–2.10)			
Formula/Mixed	227	0.76 (0.41–1.43)			
Total parenteral nutrition only	211	2.31 (1.20–4.44)			

*Confounding factors with ~10% change in OR. ELBW, extremely low birthweight; LBW, low birthweight; M, number of missing data; NBW, normal birthweight; VLBW, very low birthweight.

†Other province: Limpopo, Mpumalanga, KwaZulu-Natal, Free State, North West, Northern Cape, Western Cape, Eastern Cape. Province.

Appendix Table 7. Effect of 14 d prior exposure to antifungals on non-susceptible *Candida* sp. bloodstream infection among 1,332 old infants and older (>90 d) patients, adjusted in turn for each potential confounder, South Africa, 2012–2017

Variable	No. patients	Stratum-specific OR (95% CI)	Mantel-Haenszel summary OR (95% CI)	Score test p value	p value for test of interaction
Age group *(m = 0)**			2.51 (1.82–3.45)	<0.001	0.88
>90 d–12 mo	143	2.59 (1.11–6.05)			
>12 mo–17 y	238	1.93 (0.93–3.98)			
18–64 y	768	2.74 (1.76–4.25)			
≥65 y	193	2.43 51.02–5.76)			
Sex *(m = 1)*			2.30 (1.67–3.06)	<0.001	0.32
F	622	1.90 (1.21–2.98)			
M	719	2.60 (1.72–3.93)			
Health-sector *(m = 0)**			1.89 (1.37–2.62)	<0.001	0.33
Public	1,194	1.78 (1.28–2.50)			
Private	148	3.21 (1.02–10.13)			
Year *(m = 0)*			2.27 (1.66–3.11)	<0.001	0.34
2012–2013	351	1.55 (0.89–2.71)			
2014–2015	242	1.86 (0.81–4.27)			
2016	344	2.89 (1.37–6.13)			
2017	405	2.95 51.72–5.07)			
Province *(m = 0)*			2.12 (1.56–2.90)	<0.001	0.33
Gauteng	714	2.39 (1.06–2.86)			
Other	628	1.74 (1.60–3.56)			
ICU admission *(m = 21)*			2.14 (1.55–2.95)	<0.001	0.003
No	589	1.08 (0.62–1.86)			
Yes	742	3.03 (2.00–4.58)			
Length of hospital stay before infection onset (*m = 33)**					
≤2d	277	1.24 (0.27–5.65)	2.06 (1.50–2.83)	<0.001	0.46
3–7d	214	3.90 (1.53–9.92)			
8–14d	264	1.61 (0.80–3.24)			
15–24d	160	1.86 (1.25–6.54)			
>24d	394	1.84 (1.17–2.91)			
Prior 90d hospitalization *(m = 14)*			2.26 (1.67–3.07)	<0.001	0.51
No	286	2.83 (1.35–5.91)			
Yes	1,042	2.16 (1.54–3.02)			
HIV status *(m = 571)**			2.07 (1.38–3.10)	0.003	0.92
Seronegative	464	2.04 (1.24–3.35)			
Seropositive	307	2.13 (1.05–4.31)			
Prior antibiotics *(m = 11)*					
No	373	11.85 (0.88–3.89)	2.19 (1.61–2.98)	<0.001	0.63
Yes	958	2.26 (1.61–3.17)			

*Confounding factors with ~10% change in OR. m number of missing data

Appendix Table 8. Effect of 14 d prior-ATF exposure, grouped by molecule, on non-susceptible *Candida* sp. bloodstream infection among 1,342 older patients (>90 d) with candidaemia, unadjusted for potential confounders, South Africa, 2012–2017*

Variable	N patients exposed (%)	OR (95%CI)	p value
Prior-atf (grouped)	210 (16)	2.27 (1.68–3.07)	<0.001
Prior azole	158 (12)	1.89 (1.35–2.65)	<0.001
Prior echinocandine	43 (3)	6.75 (3.10–14.69)	<0.001
Prior amphotericine B	31 (2)	1.87 (0.91–3.84)	0.09

*Reference: no prior-exposure (N = 1,132)

Appendix Table 9. Random-effect multivariable logistic regression analysis of the effect of prior exposure to antifungals on non-susceptible *Candida* sp. bloodstream infections by potential confounder among 741 old infants and older patients with culture-confirmed candidemia, South Africa, 2012–2017*

Variable	Summary aOR for less susceptible *Candida* sp.	Wald p value
Prior antifungal exposure		
No	**Referent**	
Yes	**2.04 (1.29–3.22)**	**0.002**
Age group		0.01
Older infants (>90d–1y)	Referent	
Children – adolescents (>1y–17y)	1.22 (0.50–2.95)	0.66
Adults (18y–64y)	2.33 (1.04–5.19)	0.04
Elderly (≥65y)	2.29 (0.91–5.78)	0.08
Sex		
F	Referent	
M	1.06 (0.77–1.47)	0.7
Year		0.03
2012–2013	Referent	
2014–2015	0.88 (0.53–1.46)	0.63
2016	1.64 (1.04–2.58)	0.03
2017	1.49 (0.97–2.30)	0.07
Province		
Other	Referent	
Gauteng	1.58 (1.11–2.24)	0.01
ICU admission		
No	Referent	
Yes	1.36 (0.96–1.92)	0.08
Healthcare sector		
Private	Referent	
Public	0.25 (0.10–0.62)	0.003
Hospital stay before infection onset, d		0.87
≤2	Referent	
3–7	0.85 (0.50–1.44)	0.54
8–14	0.84 (0.52–1.39)	0.51
15–21	0.89 (0.50–1.59)	0.69
≥22	1.04 (0.65–1.67)	0.86
HIV status		
Seronegative	Referent	
Seropositive	0.62 (0.43–0.88)	0.007

*Number of cluster: 28, but with a mean obs per group of 26.5 range (1–90); Intra-cluster correlation coefficient = <0.001, p = 1
